# Longitudinal plasma protein profiling in glioblastoma patients using NULISAseq reveals treatment-associated changes across radiation and chemotherapy

**DOI:** 10.1007/s11060-026-05839-2

**Published:** 2026-10-31

**Authors:** Ashish Kumar, Yangen He, Susy Kim, Fang-Chi Hsu, Christina S. Meade, Drew D. Kiraly, Roy Strowd, Glenn J. Lesser, Gagan Deep

**Affiliations:** 1 Department of Internal Medicine, Section of Gerontology and Geriatric Medicine, Wake Forest University School of Medicine, Biotech Place, 3E-031, Winston-Salem, NC 27101 USA; 2 Department of Biostatistics and Data Science, Wake Forest University School of Medicine, Winston-Salem, NC 27157 USA; 3 Atrium Health Wake Forest Baptist Comprehensive Cancer Center, Wake Forest University School of Medicine, Winston-Salem, NC 27157 USA; 4 Translational Neuroscience, Wake Forest University School of Medicine, Winston-Salem, NC 27157 USA; 5 Department of Psychiatry, Atrium Health Wake Forest Baptist, Medical Center Boulevard, Winston-Salem, NC 27157 USA; 6 Department of Neurology, Atrium Health Wake Forest Baptist, Medical Center Boulevard, Winston-Salem, NC 27157 USA; 7 Department of Cancer Medicine, Wake Forest University School of Medicine, Winston-Salem, NC 27157 USA

**Keywords:** Glioblastoma, NULISAseq, Radiotherapy, Neuroinflammation, Alzheimer’s disease

## Abstract

**Purpose:**

Glioblastoma (GBM) is the most aggressive primary brain tumor with poor outcomes despite surgery, radiotherapy (RT), and temozolomide (TMZ). Monitoring relies on imaging, which is confounded by pseudoprogression and provides limited molecular insight. Circulating biomarkers may offer a minimally invasive approach to track disease and treatment effects.

**Methods:**

We performed targeted longitudinal proteomic profiling of plasma from post-surgery GBM patients (*n* = 11) at baseline (pre-radiotherapy (RT)), after RT with concomitant TMZ, and after adjuvant TMZ, alongside healthy controls (*n* = 15), using the NULISAseq CNS panel covering 131 proteins related to neurodegeneration, neuroinflammation, and vascular health. Differential expressions were assessed using volcano plots, hierarchical clustering, paired statistical comparisons and longitudinal trends analysis.

**Results:**

At baseline, GBM plasma showed elevated markers of neuroaxonal and glial injury, extracellular matrix regulators, and inflammatory signaling compared to controls. Several established neurodegeneration and Alzheimer’s disease- related biomarkers were also higher in GBM plasma versus controls. Following radiotherapy and concomitant TMZ, cytokines including IL10, IL5, and IL33 increased, indicating systemic immune activation, while BDNF showed a modest decline. Adjuvant TMZ treatment attenuated radiotherapy-induced cytokine elevations and reduced neuronal injury markers, suggesting therapy-driven reduction of inflammatory and neurodegenerative signals. Longitudinal analysis confirmed dynamic shifts in specific proteins across baseline, radiotherapy, and TMZ phases.

**Conclusion:**

High-sensitivity plasma proteomics suggested therapy-induced changes in CNS-specific and immune markers in GBM, highlighting candidate biomarkers that can be further developed for real-time monitoring of treatment response. These findings suggest candidate circulating markers that may complement imaging for monitoring disease biology and therapeutic response, and warrant validation in larger cohorts.

**Supplementary information:**

The online version contains supplementary material available at https://doi.org/10.1007/s11060-026-05839-2.

## Introduction

Gliomas, in particular glioblastoma (GBM), represent the most common malignant primary brain tumor within the central nervous system (CNS) and remain among the most aggressive and lethal primary malignancies worldwide. In the United States, gliomas account for approximately six cases per 100,000 individuals annually, with significant morbidity and mortality [1]. The latest World Health Organization (WHO) classification of CNS tumors categorizes a heterogeneous group of glial-derived neoplasms, including GBM, astrocytomas, and oligodendrogliomas, defined by integrated histopathological and molecular criteria. Despite incremental advances in surgical techniques and adjuvant therapies, GBM continues to be regarded as an incurable disease. Current standard-of-care consists of maximal safe surgical resection followed by radiotherapy with concomitant and adjuvant temozolomide (TMZ) chemotherapy, commonly referred to as the Stupp protocol [2]. While this multimodal approach modestly improves survival compared to radiotherapy alone, median overall survival remains approximately 14–24 months [3, 4], with a 5-year survival rate of only 10% [4, 5]. Numerous factors, including age, race, tumor location and size, hypoxia, metastasis, surgical interventions, and the interval between surgery and radiotherapy, have been shown to affect the prognosis of GBM patients [6–8]. The large variation in patient survival time underscores the urgent need for novel strategies to monitor disease and therapeutic response, and to develop a comprehensive prognostic index to enable more precise prediction of patient prognosis. In recent years, circulating biomarkers, particularly those reflecting neuroaxonal injury, neuroinflammation, and therapy-induced immunological shifts, have gained importance to complement imaging, refining risk stratification, and guide clinical decision-making in glioblastoma and other neurological disorders, including multiple sclerosis, Alzheimer’s disease, and amyotrophic lateral sclerosis [9–12].

Conventional radiotherapy along with concurrent and adjuvant TMZ is the mainstay for GBM treatment, however, no significant improvement in survival has been observed over the last 20 years [13, 14]. Substantial evidence indicated that radiotherapy could exert systemic antitumor effect by enhancing the immune activation in many tumors [15, 16]. However, radiotherapy induced cytotoxicity is a major concern as it can also remodel the tumor immune microenvironment and elicit systemic cytokine responses, which diminish immunologic activity. Increased systemic levels of cytokines is suggested to contribute to recurrence in GBM patients [17, 18]. Understanding the radiotherapy induced changes in the tumor microenvironment including, increased oxidative stress, hypoxia, neuroinflammation, changes in cell adhesion molecules, and impaired neurogenesis, can help predict the tumor recurrence at early stages [19, 20]. Similarly, TMZ, the alkylating agent integral to the Stupp protocol, exerts cytotoxic effects and can modulate inflammatory pathways [21]. Real-world and clinical trial data affirm a survival benefit when coupled with radiotherapy [2], especially when adjuvant cycles are completed; nevertheless, resistance mechanisms and systemic toxicities complicate durable control. Over 40% of patients undergoing chemotherapy and 55% of newly diagnosed cases do not benefit at all from TMZ therapy [22, 23]. GBM resistance or relapse can arise from DNA repair mechanisms, cellular survival strategies, immunosuppression, abnormal signaling pathways, and factors within the tumor microenvironment, owing to molecular heterogeneity of GBM [24–26]. As a result, novel strategies are continually being tested to improve patient survival, quality of life, and overall outcomes. Understanding therapy-induced changes in systemic immune responses and CNS-specific biomarkers may inform the rational design of combinatorial strategies, including immunotherapy, to improve clinical outcomes. In current clinical practices, longitudinal monitoring of treatment response in GBM relies on imaging modalities, such as magnetic resonance imaging (MRI). However, early detection of tumor progression by MRI remains difficult, as interpretation is confounded by pseudoprogression within the first three months after radiotherapy and by radiation necrosis occurring 3–12 months post-treatment [27, 28]. Therefore, continuous monitoring of tumor response through minimally invasive approaches offers a powerful tool to elucidate disease biology, assess therapeutic efficacy, and guide personalized treatment decisions.

Advances in ultra-sensitive proteomic platforms now enable comprehensive profiling of CNS-specific and inflammatory proteins from small plasma volumes, opening new opportunities to capture systemic signatures of GBM and its response to therapy. The NULISAseq (Nucleic acid Linked Immuno-Sandwich Assay) CNS panel, for example, profiles more than 120 targets, including microtubule-associated protein tau (MAPT) and its phosphorylated isoforms, glial fibrillary acidic protein (GFAP), neurofilament light (NfL/NEFL), and α-synuclein, from as little as 25 µL of plasma or CSF with low coefficients of variation (generally less than 10%) and high detectability. This enables broad coverage of CNS biology and dynamic measurement across several orders of magnitude. Recent large-scale studies applying NULISAseq have shown disease-specific signatures across neurodegenerative cohorts and confirmed that panels incorporating canonical CNS markers can yield clinically meaningful stratification and pathway-level insights [29–32]. These developments are directly relevant to GBM, for which traditional reliance on imaging and tissue biopsy limits monitoring frequency and may miss systemic signals of injury or treatment response. In this study, plasma samples from healthy controls and GBM patients were analyzed using the NULISAseq CNS panel covering 131 targets. Samples from GBM patients were collected at three post-surgical time points. Compared with healthy individuals, GBM patients before radiotherapy showed higher levels of proteins linked to Alzheimer’s disease signatures, neurodegeneration, and neuroinflammation. Longitudinal analysis further showed that, relative to baseline pre-radiotherapy samples, inflammatory cytokines increased after radiotherapy, while neural and glial structural and signaling proteins were reduced following chemotherapy.

## Material and methods

### Study participants

This study was conducted in accordance with the ethical principles outlined in the Declaration of Helsinki and was approved by Institutional Review Board (IRB) of Wake Forest University School of Medicine (#IRB00051088). Written informed consent was obtained from all participants prior to enrollment in the study. The clinical samples were obtained from patients seen in the neuro-oncology clinics at the Atrium Health Wake Forest Baptist Comprehensive Cancer Center in Winston-Salem, North Carolina. The 11 participants involved in this study were all diagnosed with grade 4 glioblastoma. Blood samples were collected at Atrium Health Wake Forest Baptist Hospital from post-surgery patients at different time points during treatment, including pre-radiotherapy (baseline; mean interval of 25.4 days post-surgery, range 12–39 days), post-radiotherapy (with concomitant TMZ), and post-adjuvant TMZ treatment. For comparison, plasma samples from 15 healthy control individuals (without cancer) were included. The demographic information and clinical treatment details of all participants are shown in Supplementary Table 1.

### NULISA biomarker measurements

Archived plasma samples from all participants were analyzed on the NULISAseq Argo platform using CNS disease panel 120 (ALAMAR Bioscience, Fremont, CA, USA), which measures biomarkers of neurodegeneration, inflammation, Alzheimer’s disease, and vascular health, according to manufacturer instructions [33–35]. Briefly, plasma samples were thawed at 4 °C overnight before centrifugation for 5 minutes at 2,000 ×g. Next, 25 µl of samples were transferred to the 96-well sample plate provided with the reagent kit. The plate was sealed and spun for 1 minute at 2,200 ×g and 4 °C. Target analytes within each sample were bound to a cocktail of capture and detection antibodies linked to DNA barcodes, and the resulting immunocomplex with both sample-specific and target-specific DNA tags were pooled into a single library for next generation sequencing. All samples were analyzed in the same run, with three replicates of a sample control (SC), three replicates of an inter-plate control (IPC), and four replicates of a negative control included to monitor assay performance. The library was sequenced on a NextSeq 2000 (Illumina, San Diego, CA, USA) sequencer using P2 flowcell and XLEAP-SBS™ Reagent Kit (100 Cycles) (cat# 20100987) to obtain about 400 m single-end reads for each analysis. After sequencing, the FASTQ file was uploaded to Argo Command Center (ACC) for automatic normalization and conversion to NULISA protein quantification (NPQ) units for each biomarker. NPQ was derived by dividing target counts by internal control counts of each well and then divided again by the median IPC counts, followed by log2-transformation. Differences in NPQ can be interpreted as log2(fold change). Also, the limit of detection (LOD), or the lowest concentration of the analyte that can be distinguished from the background signal of the assay, was calculated for each target as the mean plus three standard deviations of the four negative control wells normalized reads.

### Statistical analysis

Data analysis was performed using cloud-based ACC and NULISA analysis software (NAS). Volcano plots and heat maps were generated in NAS using thresholds of absolute log2 fold change ≥ 0.5 and *p* values < 0.05, without false discovery rate (FDR) correction. These visualizations are presented for exploratory purposes and hypothesis generation rather than formal hypothesis testing. To avoid excessive filtering at this stage, multiple testing correction, including FDR, correction for age and sex, was not applied for comparison with control and also for longitudinal comparisons. Box plots were generated using GraphPad Prism 10.2.1. *p*-values shown in box plots were calculated using an unpaired t-test with Welch’s correction for comparison between control and GBM baseline patients (post-surgery, pre-RT), and a paired t-test for within-subject change analysis. For comparison of AD-related biomarkers between control and GBM baseline patients, linear regression models were used with biomarkers as the outcomes and age and group (control vs. GBM) as independent variables. *p*-values for group comparisons at baseline, shown in the box plots, were obtained from these regression models. For visualization of age‑adjusted biomarkers, we extracted residuals from age‑adjusted regression models and added back the overall mean to obtain age‑adjusted values.

## Results

### Technical performance of the NULISAseq measurements

**Fig. 1:**
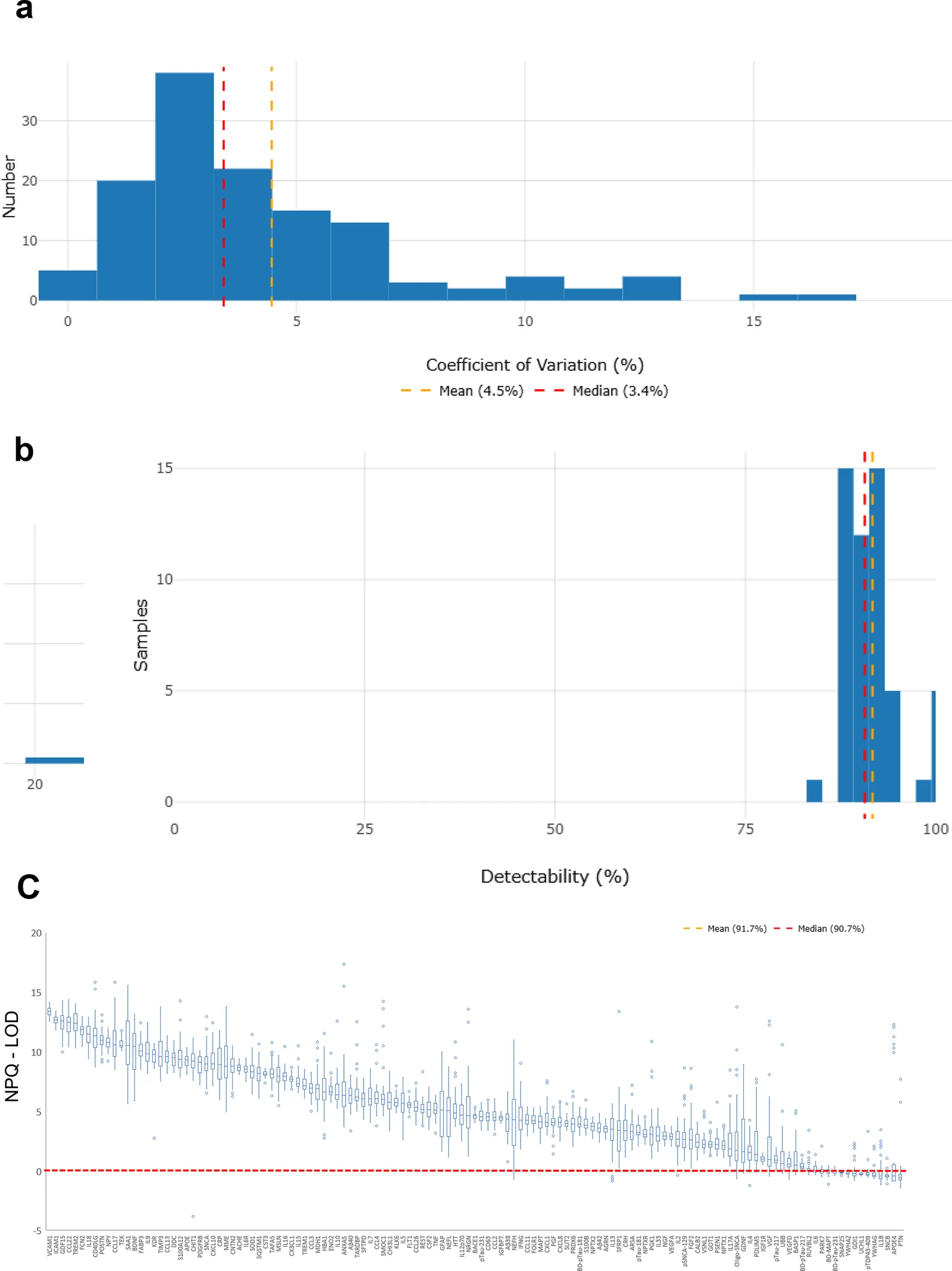
Analytical performance of NULISAseq CNS panel in plasma samples. (**a**) Histogram showing intra-plate coefficient of variation (CV, %) for targets. (**b**) Detectability (%) of the samples for all targets was analyzed by NULISA analysis software. (**c**) Box plots illustrate the detectability of 131 targets across 48 plasma samples, including longitudinally collected plasma from GBM patients (total *n* = 33). The *y*-axis displays NPQ-LOD (NULISA protein quantification-lower detection limit), where values greater than 0 indicate detectability. On each box, the central mark indicates the median, and the bottom and top edges of the box indicate the 25th and 75th percentiles, respectively

A total of 131 protein targets from all (in total 48) plasma samples were quantified using NULISAseq CNS panel in this study. All samples were analyzed on a single plate. The assay demonstrated high reproducibility and detectability with a median intra-plate mean coefficient of variation (CV) of 4.5% (Fig. 1a). Across all 48 samples, the mean detectability for all 131 targets was 91.7% (Fig. 1b), defined as the percentage of samples exceeding the limit of detection (LOD) for each target, indicating high assay sensitivity. Among all the targets proteins included in the panel (Supplementary Fig. 1), the following proteins showed expression in less than 90% of the samples: UCHL1 (ubiquitin C-terminal hydrolase L1, 0.0%), SNCB (beta-synuclein, 4.2%), YWHAG (14–3–3 gamma, 8.3%), PTN (pleiotrophin, 12.5%), APOE4 (apolipoprotein E 4, 33.3%), IL1B (interleukin-1 beta, 22.9%), p-TDP43 (phosphorylated- TAR DNA-binding protein 43, 16.7%), GDI1 (GDP-dissociation inhibitor 1, 27.1%), SNAP-25 (synaptosomal-associated protein, 25 kDa, 4.2%), YWHAZ (14–3-3ζ, 14.6%), BD-pTau-231 (brain-derived phosphorylated Tau-231, 47.9%), IL6 (interleukin 6, 56.2%), BD-MAPT (brain-derived microtubule-associated protein, 47.9.8%), PARK7 (parkinson disease protein 7, 34.4%), and RUVBL2 (RuvB Like AAA ATPase 2, 70.8%) (Fig. 1c). The target detectability plot shows target NPQ distributions relative to LOD. For each target, LOD in NPQ units was subtracted from NPQ (Fig. 1c).

### Targeted proteomics profiling in plasma from GBM patients compared to controls

**Fig. 2:**
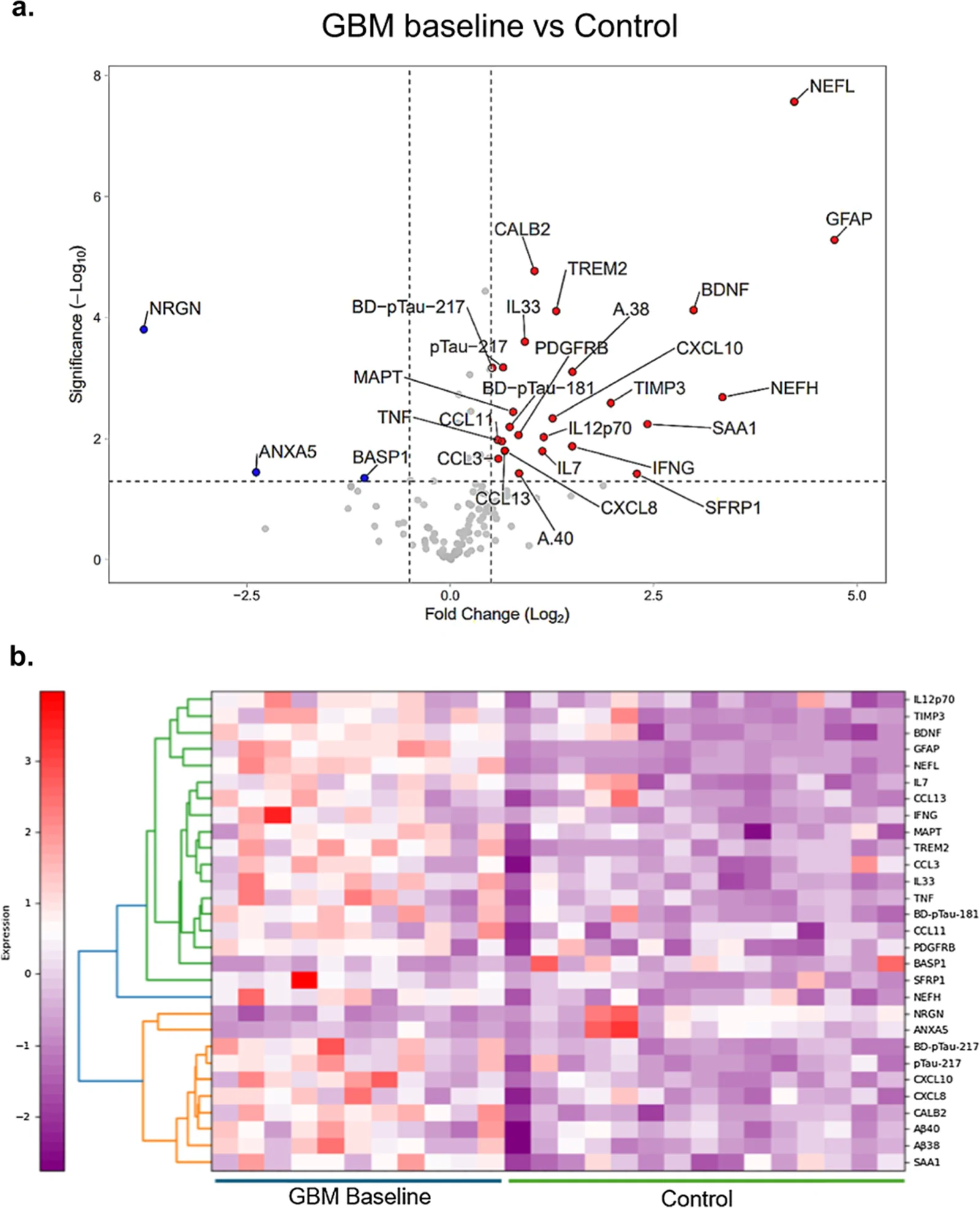

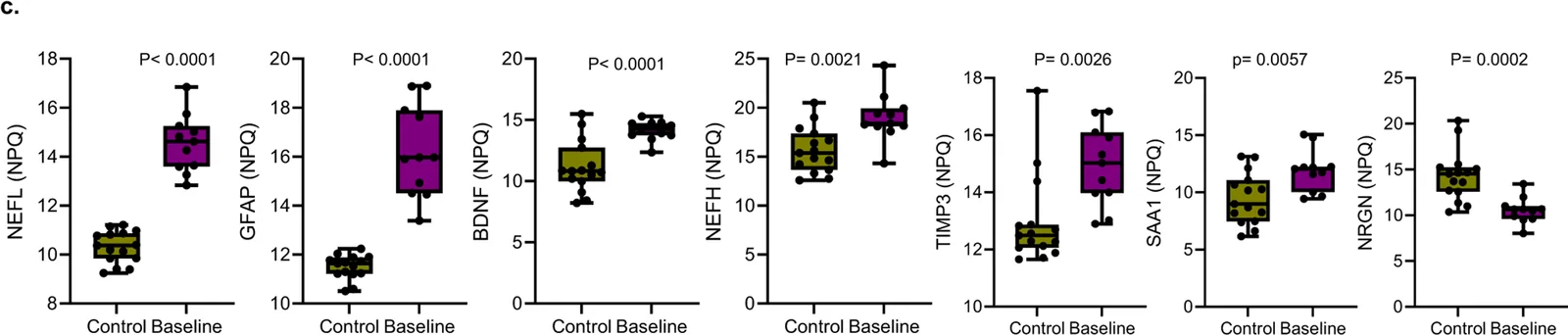
Differential plasma proteomic signatures in GBM patients at baseline compared to controls. Targeted proteomics profiling of plasma from GBM patients (*n* = 11) at baseline (post-surgery before radiotherapy) and non-cancer controls (*n* = 15) individuals using NULISAseq CNS panel. (**a**) Volcano plot represents the differentially expressed proteins between GBM-baseline vs controls. Y-axis represents the -Log10 *p*-value and X-axis shows the Log2 Fold change. Red dots represent the upregulated proteins while blue dots represent downregulated proteins in the plasma of GBM-baseline (Log2 -0.5 to 0.5, and *p* < 0.05). (**b**) Hierarchical clustering heatmap of normalized protein expression (Z-scores) for all 131 proteins profiled. Rows represent proteins, columns represent individual samples. Color scale indicates relative abundance (red = higher, blue = lower) (**c**) Box plots of selected significantly altered proteins comparing GBM baseline and controls: NEFL, GFAP, BDNF, NEFH, TIMP3, SAA1, and NRGN. Data is shown as normalized NULISA protein quantification (NPQ). Unpaired t-test with Welch’s correction were used to calculate statistical significance (*p* < 0.05). *p* values are shown on the box plots

To investigate systemic protein alterations associated with GBM, we profiled plasma samples from GBM patients at baseline (post-surgery and pre-RT) and from control individuals (without cancer) using the NULISAseq panel to profile 131 CNS related proteins. Volcano plot analysis revealed multiple proteins significantly dysregulated in GBM patients compared to controls (Fig. 2a). Notably, neurofilament light (NEFL) and neurofilament heavy chain (NEFH), glial fibrillary acidic protein (GFAP), brain-derived neurotrophic factor (BDNF), serum amyloid A1 protein (SAA1), and tissue inhibitor of metalloproteinases-3 (TIMP3) were among the most upregulated proteins in GBM plasma. In addition, the levels of several chemokines, cytokines, and inflammatory mediators, including CXCL10 (C-X-C motif chemokine ligand 10), CXCL8 (C-X-C motif chemokine ligand 8), CCL3 (C-C motif chemokine ligand 3), CCL11 (C-C motif chemokine ligand 11), IL33, IL7, and IL12p70, were also found to be higher in the plasma of GBM patients compared to controls. Importantly, several typical Alzheimer’s disease related markers such as brain-derived (BD)-ptau-217, BD-pTau-181, pTau-217, amyloid beta (Aβ)-42, and Aβ-38 also showed statistically significantly higher levels in GBM baseline patients. In contrast, a strong and significant reduced level of neurogranin (NRGN) was observed along with marginally lower levels of annexin A5 (ANXA5), and the brain abundant membrane attached signal protein 1 (BASP1) in the plasma of GBM patients (Fig. 2a). Hierarchical clustering of normalized NULISA protein quantification (NPQ) values for the significantly differentially expressed proteins, demonstrated clear segregation between GBM baseline and control samples (Fig. 2b). The most significantly altered proteins (baseline glioma versus control) are also shown as box plots in Fig. 2c.

**Fig. 3:**
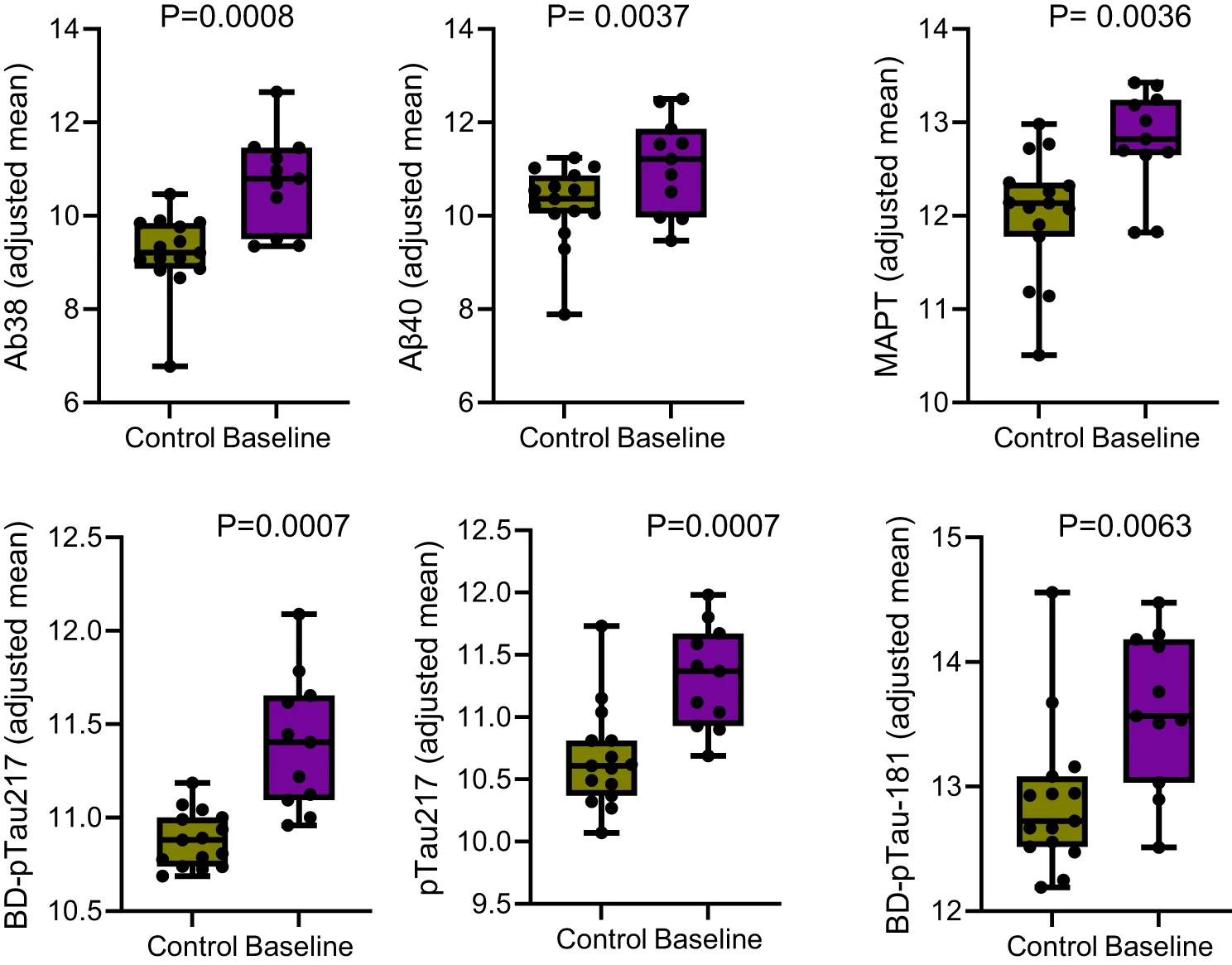
Age-adjusted analysis of Alzheimer’s disease-related biomarkers in GBM plasma. Analysis of Alzheimer’s disease (AD)-related biomarkers in the plasma of GBM-baseline (*n* = 11) and control individuals (*n* = 15). The NPQ values from healthy controls and GBM patients were adjusted for age and box plots were generated. Y-axis shows age adjusted NPQ values. Unpaired t-test with Welch’s correction were used to calculate statistical significance. *p* values are shown on the box plots

Since Alzheimer’s disease-related markers can be affected by age, age-adjustment was performed, and GBM patients still showed significantly higher levels of several well‑established AD-related biomarkers, including Aβ38 (*p* = 0.0008), Aβ40 (0.0037), MAPT (*p* = 0.0036), brain-derived (BD)-pTau217 (*p* = 0.0007), pTau217 (*p* = 0.0007), and BD-pTau181 (*p* = 0.0063) (Fig. 3).

### Radiotherapy with concomitant temozolomide-induced proteomics changes in the plasma of GBM patients

**Fig. 4:**
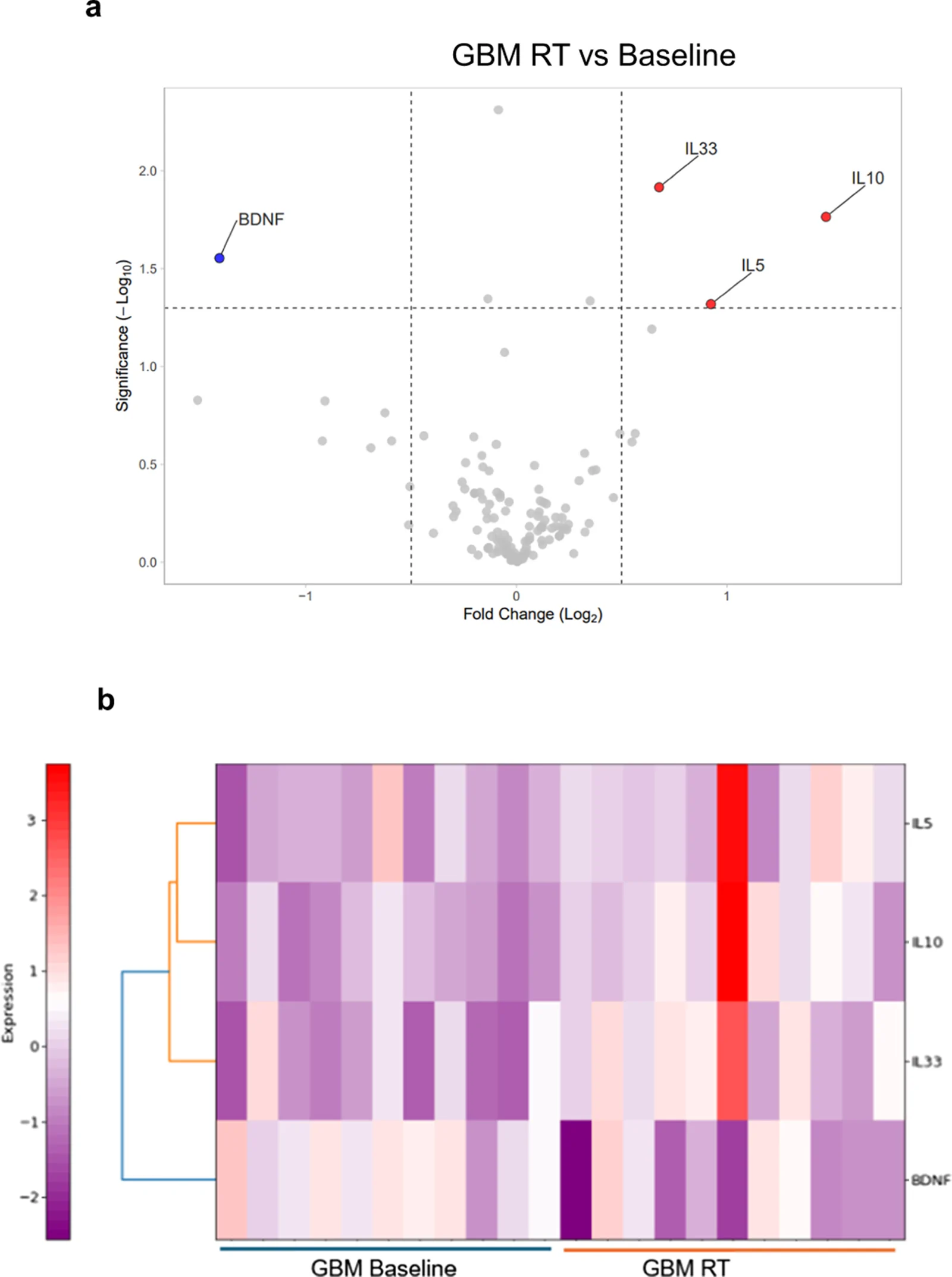

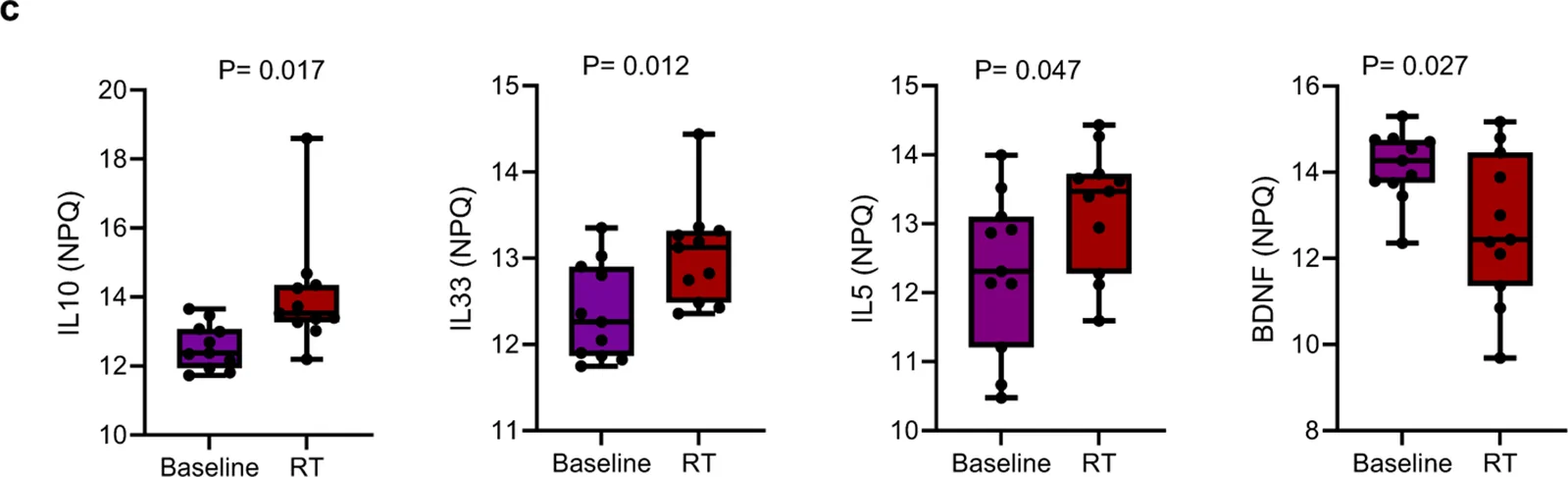
Plasma proteomic changes in GBM patients following radiotherapy with concomitant temozolomide (TMZ) compared to baseline. (**a**) Volcano plot illustrates differential protein expression between paired GBM samples collected after radiotherapy (RT) with concomitant TMZ and at baseline (*n* = 11/ each, paired). Y-axis represents the -Log10 *p*-value and X-axis shows the Log2 Fold change. Red dots represent the upregulated proteins while blue dots represent downregulated in the plasma of GBM-radiotherapy (Log2 -0.5 to 0.5, and *p* < 0.05). (**b**) Heatmap showing hierarchical clustering of normalized NPQ (Z-scores) for differentially expressed proteins. Columns represent individual samples, and rows represent proteins. Color scale indicates relative abundance (red = higher, blue = lower) c. box plots of selected significantly altered proteins comparing baseline and radiotherapy samples: IL10, IL5, IL33, and BDNF. Data are shown as normalized NPQ. *p*-values were calculated using paired statistical tests (*p* < 0.05). *p* values are shown on the box plots

To assess radiation treatment-associated alterations in circulating proteins, we compared plasma samples for protein expression from the same GBM patients profiled at baseline and after completion of radiotherapy along with concomitant TMZ. Volcano plot analysis revealed modest but significant changes in immune-related proteins following radiotherapy (Fig. 4a). Interleukins-10 (IL10), IL5, and IL33, showed significant higher levels whereas the level of BDNF showed lower level in GBM patients after radiotherapy plasma compared to baseline. Hierarchical clustering of normalized NPQ values of the significantly altered proteins demonstrated partial segregation of radiotherapy samples from baseline samples (Fig. 4b). Box plot analysis confirmed significant differences for selected proteins between baseline and radiotherapy samples (Fig. 4c). IL10 (*p* = 0.017), IL33 (*p* = 0.012), and IL5 (*p* = 0.047), were significantly elevated after radiotherapy, while BDNF (*p* = 0.027) demonstrated wider distribution within radiotherapy group but showed overall reduced levels compared to baseline (Fig. 4c).

Interestingly, compared to control healthy individuals (without cancer), GBM patients after radiotherapy continue to exhibit elevated levels of proteins that were already increased at baseline. NEFL, GFAP, NEFH, IL33, TREM2, SAA1 along with other neurodegeneration and neuroinflammation related proteins showed higher levels, while NRGN and huntingtin (HTT) showed lower levels after radiotherapy compared to controls. Importantly, the levels of AD-related markers, including MAPT, Aβ38, Aβ40, and BD-pTau-181 also showed elevated levels after radiotherapy along with concomitant TMZ compared to controls (Supplementary Fig. 2a). Moreover, differentially expressed proteins after radiotherapy showed distinct clustering compared to controls (Supplementary Fig. 2b).

### Plasma proteomic changes following adjuvant TMZ chemotherapy

**Fig. 5:**
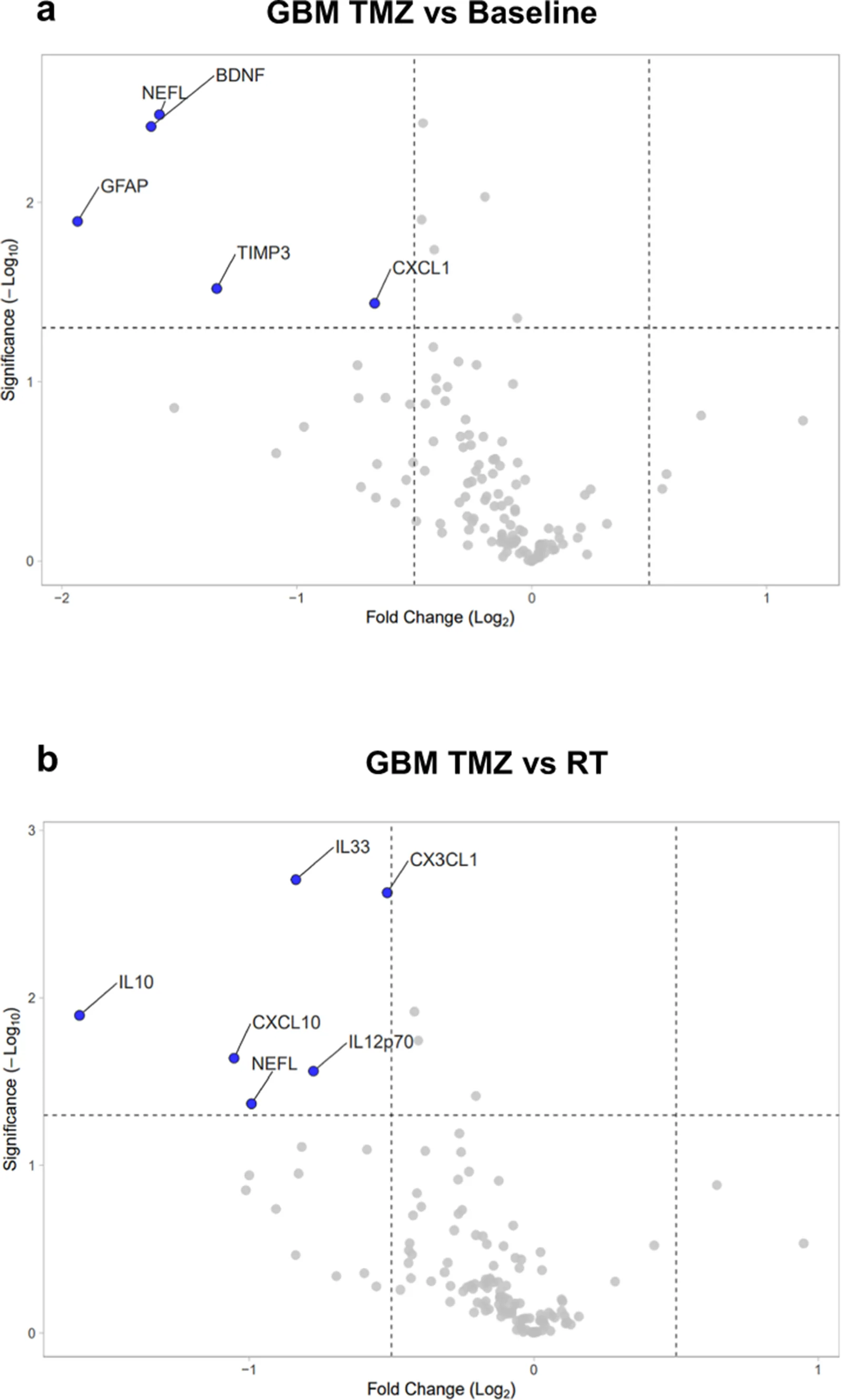

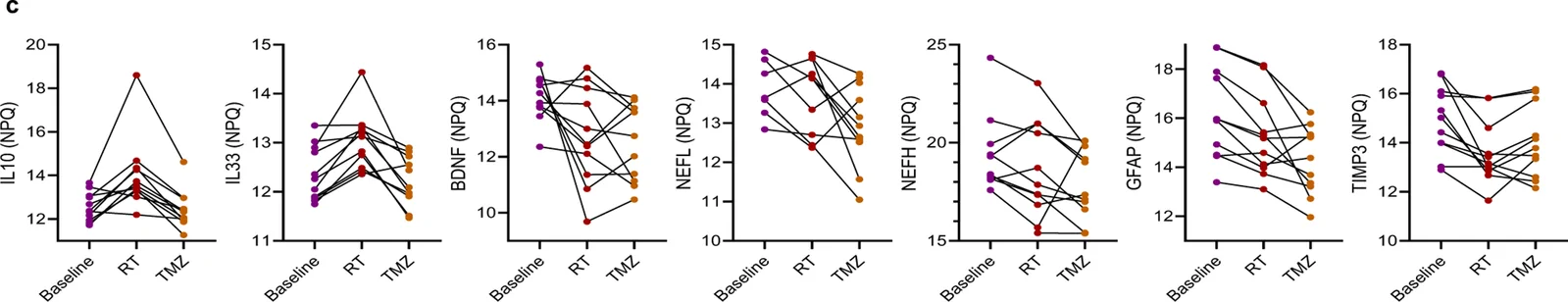
Plasma proteomic changes in GBM patients following adjuvant temozolomide (TMZ) chemotherapy compared to baseline or post-radiotherapy with concomitant TMZ. Volcano plot showing differential protein expression between paired GBM samples collected after adjuvant TMZ treatment and at baseline (*n* = 11/ each, paired) (**a**) or after radiotherapy with concomitant TMZ (**b**). Y-axis represents the -Log10 *p*-value and X-axis shows the Log2 Fold change. Each point represents a protein; blue indicates proteins significantly downregulated after TMZ (Log2 -0.5 to 0.5, and *p* < 0.05). c. line plots showing longitudinal changes in selected proteins across three time points, baseline, radiotherapy (RT) with concomitant TMZ, and adjuvant TMZ for each patient

To evaluate the impact of adjuvant TMZ treatment on circulating protein profiles, we analyzed plasma samples from the same GBM patients after adjuvant chemotherapy (TMZ) and compared them to their baseline and post-radiotherapy with concomitant TMZ samples. Volcano plots illustrate the changes in protein abundance after adjuvant TMZ relative to baseline (Fig. 5a) and radiotherapy with concomitant TMZ (Fig. 5b). Interestingly, compared to the baseline, the levels of NEFL, BDNF, GFAP, TIMP3, and CXCL1 showed lower levels post-adjuvant TMZ treatment. As compared to RT with concomitant TMZ, adjuvant TMZ treatment was associated with decreased levels of inflammatory cytokines such as IL10, IL33, Il12p70, CX3CL1, and CXCL10. The levels of NEFL were also found to be reduced after adjuvant TMZ treatment compared to RT with concomitant TMZ.

To visualize longitudinal trends, line plots were generated for selected proteins across baseline, RT, and TMZ time points (Fig. 5c). IL10 and IL33 showed marked increases after RT, followed by a decline in post-TMZ. BDNF, NEFL and NEFH, as well as GFAP and TIMP3, demonstrated progressive reductions from baseline to TMZ (Fig. 5c).

However, compared to control individuals, post-adjuvant TMZ treatment continued to show higher levels of NEFL, GFAP, IL33, TREM2, and NEFH, while the NRGN showed reduced levels. Moreover, the levels of MAPT and pTau did not show any significant change between post-adjuvant TMZ and controls, while Aβ38 and Aβ40 retained higher levels (Supplementary Fig. 3a). Furthermore, the heatmaps showed distinct clustering between post-TMZ and control groups (Supplementary Fig. 3b).

## Discussion

Monitoring biomarkers of inflammation and neurodegeneration in the plasma of GBM patients “during and after” treatment could be valuable to predict changes in the tumor microenvironment and offer valuable information about treatment effect. The standard of practice for GBM evaluation is by histopathological examinations and molecular characterization by tissue biopsy. This approach is typically limited to an initial surgical resection or biopsy and is generally not feasible to perform repeated assessments during disease progression or treatment response. Currently, GBM has no blood-based marker that can accurately diagnose, stage or monitor response to treatment modalities, or recurrence [36, 37]. Moreover, assessment of blood-based markers in the real-world setting is limited because of several challenges, including the limited release of brain tumor-derived biomarkers across the blood-brain barrier into the circulation. In addition, the rate of shedding throughout the disease course and the influence of tumor characteristics, such as location, size, histology, grade, and amount of enhancing versus non-enhancing disease, remain to be addressed [36]. Sensitive detection of modest signals from GBM in blood is required to effectively capture and draw some meaningful information about the tumor response following therapy. High-sensitivity, multiplexed proteomic platforms, including NULISAseq, have transformed the feasibility of plasma biomarker discovery for brain diseases by reaching the attomolar sensitivity (10^−18^ moles per liter) and achieving robust detectability across neuro-specific and inflammatory proteins.

Utilizing the NULISAseq CNS panel, we interrogated the plasma proteome of GBM patients across the canonical treatment trajectory, baseline (post-surgery, pre-radiotherapy), post-radiotherapy with concomitant TMZ, and post-adjuvant TMZ, and contrasted these time points with control individuals (without cancer). At baseline, we observed a distinct GBM-associated signature characterized by differential levels in the markers of axonal and astroglial injury (NEFL, NEFH, GFAP), synaptic and neurotrophic pathways (BDNF, NRGN), and extracellular matrix remodeling (TIMP3), along with several inflammatory mediators (e.g., CXCL8, CXCL10, CCL3, IL33, IL7, TNF). These changes are consistent with prior literature showing that GFAP and NEFL/NEFH increase in the setting of active CNS pathology and that chemokines contribute to GBM progression and immunosuppression via macrophage recruitment, angiogenesis, and epithelial-to-mesenchymal (EMT)-like transitions [38–41]. In line with these findings, elevated plasma levels of GFAP, NFL, and Tau have been reported in GBM patients compared to controls. Also, GFAP has been reported to demonstrate the strongest individual diagnostic power, with an AUC of 0.99 and nearly complete group separation [42]. GFAP has been shown to serve as a biomarker for tumor progression and treatment response in various cancers including GBM and astrocytoma [43]. Notably, we detected higher levels of typical Alzheimer’s disease biomarkers in the plasma of GBM patients compared to controls in our cohort. Tau, the protein product of the MAPT gene, has shown altered transcription and expression across several cancers, including breast, prostate, gastric, ovarian, pediatric neuroblastoma, and gliomas. Elevated Tau expression has been associated with resistance to microtubule‑targeting agents, such as taxanes, particularly in breast and ovarian cancers [44–46]. During tumor growth, GBM cells have been reported to secrete a soluble form of CD44 which induced neuronal degradation via activation of tau pathology in the brain [47]. Conversely, higher tau levels have also been associated with isocitrate dehydrogenase (IDH1) mutations in glioma and positively correlated with improved survival independent of therapy in breast cancer and low‑grade glioma, underscoring the context‑dependent role of Tau in cancer biology [48–50]. Tau proteins inside and outside neurons have been reported to interact with cancer-related kinases and are known to affect a diverse range of cellular pathways including cell cycle, cell migration, angiogenesis, cellular metabolism, genomic modifications, and they have been suggested as novel therapeutic targets for GBM [51].

Similarly, accumulation of amyloid beta (Aβ) peptides has been reported in glioma, where amyloid deposition is localized to the tumor microenvironment and associated with glioma cells and tumor vasculature, rather than reflecting a generalized systemic increase in Aβ production [52]. However, peripheral sources such as hyperactive platelets, which are also common in cancer, can also contribute to circulating Aβ following thrombosis [53]. We detected elevated levels of Aβ isoforms in the plasma of GBM patients, although the relative contribution of tumor‑derived versus peripheral sources remains unclear. Amyloid‑related peptides can engage both activating and inhibitory immune pathways, and their context‑dependent immunomodulatory effects are increasingly being recognized in cancer biology [54, 55]. Elevated circulating Aβ38 and phosphorylated tau in GBM patients may therefore reflect a broader interplay between CNS injury, innate immune activation, and tumor‑associated amyloid signaling. Moreover, the accumulation of these Alzheimer’s disease-related proteins (amyloid and Tau), especially the brain-derived total Tau (MAPT) and phospho-tau isoforms, not only indicates neurodegeneration but also suggests a high risk for cognitive impairments in GBM patients.

Further, there is evidence to suggest that higher levels of SAA1 correlate directly with poor prognosis in various systemic cancers such as lung, breast, melanoma, gastric, prostate, esophageal, endometrial and renal [55, 56]. Chemokines and cytokines, including CXCL8, CXCL10, TNF, IL-10, IL-15, and IL-33, mediate tumor immune interactions and are dynamically regulated by therapy; they influence angiogenesis, macrophage polarization, effector T-cell function, and the immunogenic balance within the tumor microenvironment [41, 57–59]. In GBM, CXCL8 promotes a mesenchymal tumor state and supports M2-like macrophages, whereas CXCL10 produced by glioma cells can drive proliferative signaling through ERK1/2, highlighting chemokines and their downstream signaling as potential biomarkers and therapeutic targets [40, 60, 61]. Neurotrophic factors such as BDNF further integrate neuronal plasticity with disease biology. For example, meta-analyses indicated altered peripheral BDNF levels in glioma, and with a known role of BDNF in neuroprotection and synaptic remodeling, therapy-induced BDNF changes could reflect adaptive CNS responses [62, 63]. Microglial and macrophage receptors such as TREM2, also showed higher levels at baseline, further illustrating the interplay between neuroinflammation and glioma biology.

Radiotherapy is increasingly recognized not only for its direct cytotoxicity but also for its capacity to remodel the tumor immune microenvironment and elicit systemic cytokine responses. Experimental and clinical observations describe a surge of pro- and anti-inflammatory cytokine release after ionizing radiation. In some settings, radiation can induce immunogenic cell death and promote dendritic cell engagement, while in others feedback circuits can enforce immune tolerance [64, 65]. Consistent with these concepts, our paired analyses in the same individuals demonstrated post-radiotherapy with concomitant TMZ increases in IL-10, IL-5, and IL-33. IL-5 regulates eosinophil differentiation, recruitment, and survival and may influence the tumor immune microenvironment through eosinophil-mediated immune responses [66, 67]. Whereas IL-10 and IL-33 can be induced as regulatory signals that temper inflammatory injury and shape macrophage- regulatory T-cells (Treg) crosstalk [68]. Notably, we also observed reduced levels of BDNF after radiotherapy, which may reflect neurotrophic responses to radiation-induced stress or inflammatory signals. Independent evidence identified BDNF as a key regulator of neural plasticity and survival, with altered BDNF signaling implicated in several neurological disorders [63]. Importantly, radiotherapy is given concomitantly with TMZ, the observed increase in the immune response and reduced BDNF reflects combined chemoradiation exposure rather than radiation alone.

TMZ, a DNA alkylating agent, exerts cytotoxic effects and can modulate inflammatory pathways. Real-world and trial data affirm its survival benefit when coupled with radiotherapy, especially when adjuvant cycles are completed; nevertheless, resistance mechanisms and systemic toxicities may complicate its long-term use [69–71]. Prolonged therapy leads to TMZ resistance and poor responsiveness mainly due to induction of enhanced levels of DNA damage repair enzymes; thus, leading to no survival benefits in 60–70% of patients [72–74]. Furthermore, multiple studies have found that treatment with radiation and TMZ induce lymphopenia (lower levels of lymphocytes), though without compensatory increases in cytokines (such as IL-7 and IL-15) which are required for lymphocyte proliferation [75–78]. We observed a moderate but statistically significant reduction in plasma IL-33 and IL-10 levels following adjuvant TMZ treatment compared to radiotherapy with concomitant TMZ, underscoring the utility of plasma protein profiling in understanding treatment-induced immune modulation. These findings highlight that systematic monitoring of circulating cytokines provides actionable insights for tailoring therapeutic approaches and optimizing outcomes in GBM patients. Furthermore, in our cohort, adjuvant TMZ was associated with attenuation of the radiotherapy with concomitant TMZ-induced cytokine surge (declines in IL-10, IL-33, IL12p70, CXCL1, CXCL10) and reductions in neuronal/glial injury marker (NEFL, GFAP) and TIMP3 relative to baseline and post-RT, implying a shift toward decreased inflammatory signaling and potential normalization of injury biomarkers with chemotherapy. These longitudinal patterns align with reports that chemoradiation alters circulating chemokine profiles, e.g., changes in CXCL10 and related members, and that systemic cytokine milieu can evolve across therapy phases in ways that reflect tumor-host dynamics [79]. However, a potential consideration when interpreting baseline biomarker elevations and subsequent longitudinal declines is the effect of surgical intervention. Surgical resection or biopsy induces acute neuroaxonal and glial trauma, leading to the transient release of structural proteins such as NEFL, and NEFH into circulation [9]. In our cohort, baseline plasma samples were collected at a mean of 25.4 days (range: 12–39 days) following surgery prior to radiotherapy initiation (Supplementary Table 1). Consequently, baseline elevations in these neuronal injury markers may reflect a combination of active tumor-associated pathology and residual perioperative tissue injury resolution. Nevertheless, elevated levels of these markers persisted significantly above non-cancer control levels following both RT and TMZ treatments (Supplementary Fig. 2a and 3a), suggesting ongoing disease- and treatment-associated CNS alterations beyond acute surgical injury resolution alone. Moreover, the inflammatory cytokine changes following radiotherapy with concomitant TMZ treatment, might be, at least in part, contributed by simultaneous low dose exposure to dexamethasone.

Collectively, our results support a working model in which plasma proteomics can (i) distinguish GBM patients from non-cancer controls, with higher levels of canonical injury and neuroinflammatory markers; (ii) capture radiotherapy with concomitant TMZ-associated immune modulation, including surges in regulatory and stimulatory cytokines; and (iii) track adjuvant TMZ-related dampening of inflammatory signals alongside reductions in neuronal/glial injury proteins. These findings extend the promise of high-sensitivity assays like NULISAseq for serial, low-volume monitoring across treatment milestones, leveraging wide dynamic range and multi-analyte coverage to map CNS disease biology from blood. The translational implications of our study are twofold. First, composite plasma signatures may complement imaging to identify therapy-responsive biology, e.g., declining GFAP/NfL/NEFH and chemokine/cytokine normalization, potentially distinguishing anti-tumor effects from treatment-related injury [38]. Second, pathway-anchored targets (CXCL8/CXCL10 axis, IL-5/IL-33 circuits, TREM2-linked microglial programs, TIMP3-mediated matrix remodeling) may guide biomarker-based stratification in trials that seek to optimize chemoradiation timing or add immune modulators to improve outcomes beyond the traditional therapeutic modality. However, multiple testing for age and sex correction along with FDR corrections were not applied for the comparisons with non-cancer controls and also for longitudinal comparisons to avoid overly stringent filtering at this exploratory stage, to rule out false‑negative findings, these findings should be interpreted cautiously and should be validated in independent larger cohorts.

While our study focuses on a limited number of time points within the standard care continuum, it illustrates that a targeted CNS proteomic panel can reveal complex biological narratives in plasma, such as injury and inflammation at baseline; radiotherapy-induced immune reprogramming; and chemotherapy-associated modulation. Future work should expand cohort size, integrate orthogonal modalities (e.g., imaging metrics, MGMT methylation status, and IDH1 profiling), and test whether composite plasma indices predict clinical endpoints such as progression-free or overall survival, objectives aligned with ongoing efforts to refine current tumor management and to translate biomarker-informed immunomodulation into practice. Ultimately, the convergence of sensitive proteomics and longitudinal sampling may enable truly dynamic monitoring of GBM biology and treatment response in routine care.

## Electronic supplementary material

Below is the link to the electronic supplementary material.

Supplementary Material 1

Supplementary Material 2

Supplementary Material 3

Supplementary Material 4

## Data Availability

The datasets generated during the current study are available from the corresponding author on reasonable request.
