# Supplementary material for: Longitudinal plasma protein profiling in glioblastoma patients using NULISAseq reveals treatment-associated changes across radiation and chemotherapy
Source: J Neurooncol. 2026 Oct 31;180(2):33. doi: 10.1007/s11060-026-05839-2 (PMC13723690; doi:10.1007/s11060-026-05839-2)
Supplement: Supplementary file 1 [file 11060_2026_5839_MOESM1_ESM.pdf]

ACHE	CCL22	FGF2	IL1B	NPY	SNAP25	pSNCA-129
AGRN	CCL26	FLT1	IL2	NRGN	SNCA	pTDP43-409
ANXA5	CCL3	FOLR1	IL33	Oligo-SNCA	SNCB	pTau-181
APOE	CCL4	GDF15	IL4	PARK7	SOD1	pTau-217
APOE4	CD40LG	GDI1	IL5	PDGFRB	SQSTM1	pTau-231
ARSA	CD63	GDNF	IL6	PDLIM5	TAFA5	CCL2
Aβ38	CHI3L1	GFAP	IL6R	PGF	TARDBP	FCN2
Aβ40	CHIT1	GOT1	IL7	PGK1	TEK	IL18
Aβ42	CNTN2	HBA1	IL9	POSTN	TIMP3	NPTXR
BACE1	CRH	HTT	KDR	PRDX6	TNF	SMOC1
BASP1	CRP	ICAM1	KLK6	PSEN1	TREM1	YWHAZ
BD-MAPT	CSF2	IFNG	MAPT	PTN	TREM2	CCL17
BD-pTau-181	CST3	IGF1R	MDH1	REST	UBB	FABP3
BD-pTau-217	CX3CL1	IGFBP7	MME	RUVBL2	UCHL1	IL17A
BD-pTau-231	CXCL1	IL10	MSLN	S100A12	VCAM1	NPTX2
BDNF	CXCL10	IL12p70	NEFH	S100B	VEGFA	SLIT2
CALB2	CXCL8	IL13	NEFL	SAA1	VEGFD	YWHAG
CCL11	DDC	IL15	NGF	SFRP1	VGF	
CCL13	ENO2	IL16	NPTX1	SFTPD	VSNL1	

Supplementary Figure 1. List of the proteins included in NULISA CNS panel.
