# Supplementary material for: Longitudinal plasma protein profiling in glioblastoma patients using NULISAseq reveals treatment-associated changes across radiation and chemotherapy
Source: J Neurooncol. 2026 Oct 31;180(2):33. doi: 10.1007/s11060-026-05839-2 (PMC13723690; doi:10.1007/s11060-026-05839-2)
Supplement: Supplementary file 2 [file 11060_2026_5839_MOESM2_ESM.pdf]

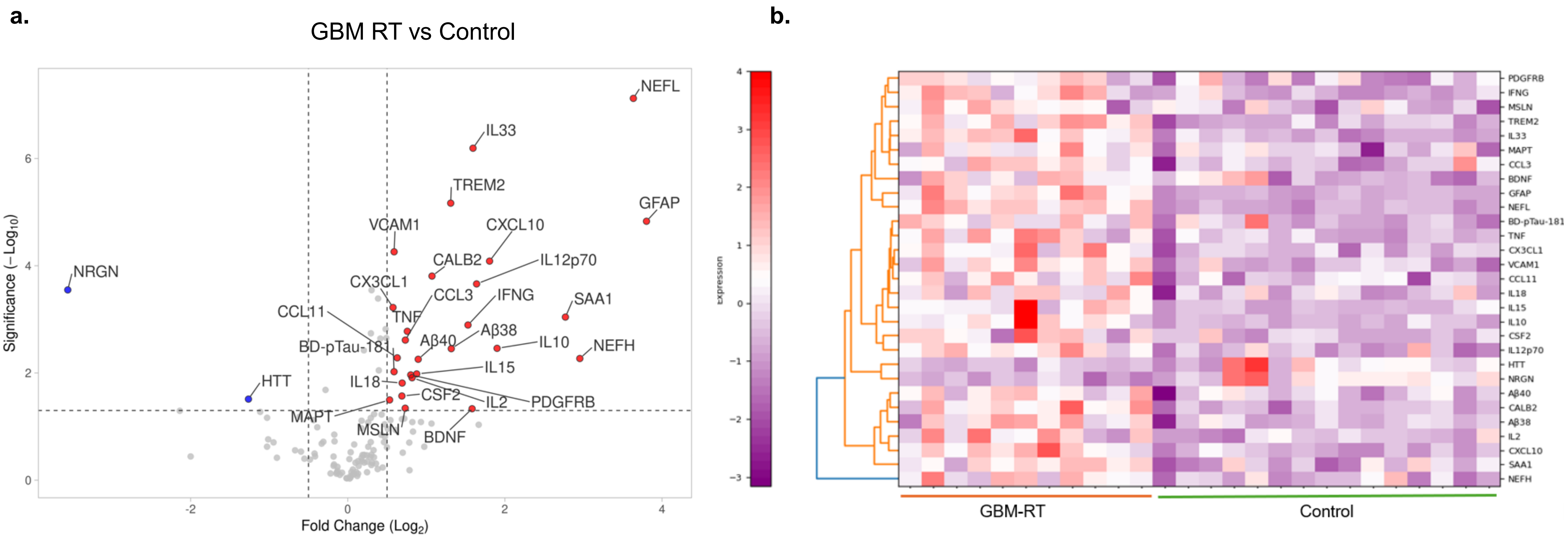

Supplementary Figure 2. Proteomic differences between post-radiotherapy with concomitant TMZ GBM plasma and control plasma samples. a. Volcano plot illustrating differential protein expression between plasma of GBM patients collected after radiotherapy with concomitant TMZ (RT, n=11) and plasma of control individuals without cancer (n=15). Y-axis represents the -Log10 p-value and X-axis shows the Log2 fold change. Red dots represent the upregulated proteins while blue dots represent downregulated proteins in the plasma of GBM-RT (Log2 -0.5 to 0.5, and p< 0.05). **b.** Heatmap showing hierarchical clustering of normalized NPQ expression (Z-scores) for differentially expressed proteins. Columns represent individual samples, and rows represent proteins. Color scale indicates relative abundance (red=higher, blue=lower).
