# Supplementary material for: Longitudinal plasma protein profiling in glioblastoma patients using NULISAseq reveals treatment-associated changes across radiation and chemotherapy
Source: J Neurooncol. 2026 Oct 31;180(2):33. doi: 10.1007/s11060-026-05839-2 (PMC13723690; doi:10.1007/s11060-026-05839-2)
Supplement: Supplementary file 3 [file 11060_2026_5839_MOESM3_ESM.pdf]

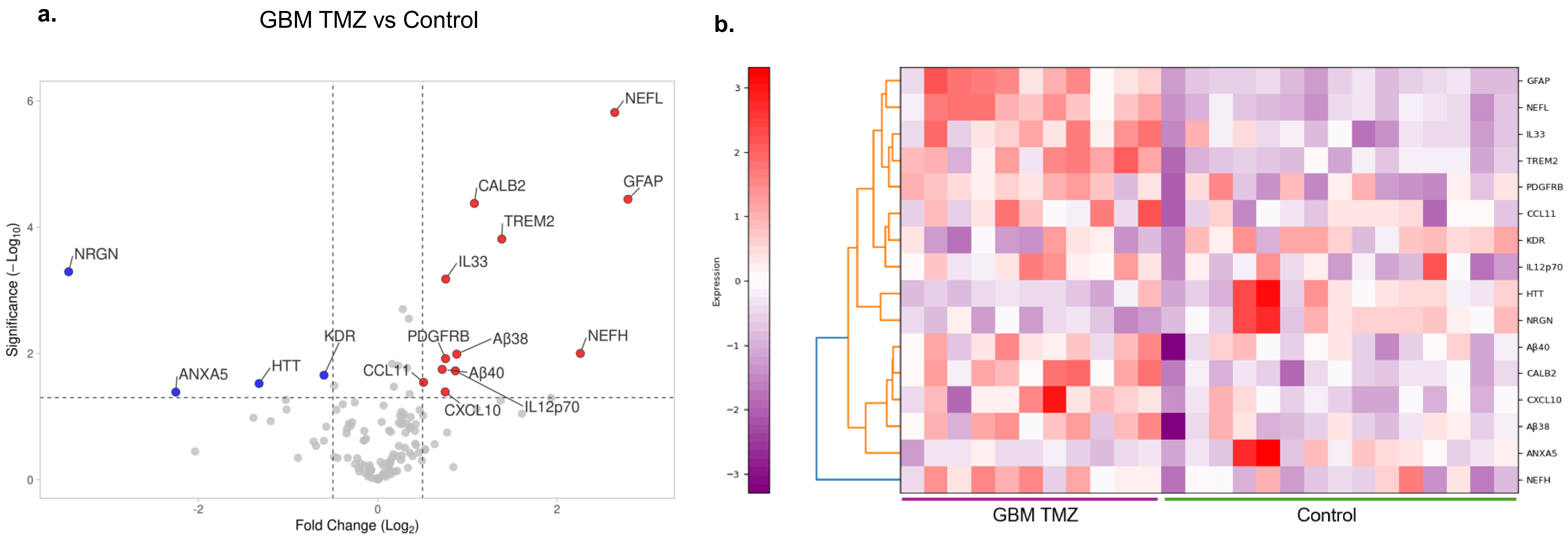

Supplementary Figure 3. Proteomic differences between post-adjuvant temozolomide GBM plasma and controls. a. Volcano plot illustrating differential protein expression between plasma of GBM patients collected after adjuvant TMZ chemotherapy (n=11) and plasma of control individuals (n = 15). Y-axis represents the -Log10 p-value and X-axis shows the Log2 fold change. Red dots represent the upregulated proteins while blue dots represent downregulated in the plasma of GBM-TMZ (Log2 -0.5 to 0.5, and p< 0.05). **b.** Heatmap showing hierarchical clustering of normalized NULISA protein expression (Z-scores) for differentially expressed proteins. Columns represent individual samples, and rows represent proteins. Color scale indicates relative abundance (red = higher, blue = lower)
